# The Effects of Baduanjin Qigong on Postural Stability, Proprioception, and Symptoms of Patients With Knee Osteoarthritis: A Randomized Controlled Trial

**DOI:** 10.3389/fmed.2019.00307

**Published:** 2020-01-10

**Authors:** Jiajia Ye, Michael William Simpson, Yang Liu, Wei Lin, Weihong Zhong, Shuhe Cai, Liye Zou

**Affiliations:** ^1^Department of Rehabilitation Assessment, Rehabilitation Hospital Affiliated to Fujian University of Traditional Chinese Medicine, Fuzhou, China; ^2^Department of Rehabilitation Sciences, The Hong Kong Polytechnic University, Hong Kong, China; ^3^Department of Kinesiology and Program in Neuroscience, Indiana University Bloomington, Bloomington, IN, United States; ^4^Academy of Integrative Medicine, Fujian University of Traditional Chinese Medicine, Fuzhou, China; ^5^Department of Orthopaedic Rehabilitation, Rehabilitation Hospital Affiliated to Fujian University of Traditional Chinese Medicine, Fuzhou, China; ^6^Fujian Provincial Rehabilitation Industrial Institution, Fuzhou, China; ^7^Exercise and Mental Health Laboratory, Shenzhen Key Laboratory of Affective and Social Cognitive Science, Shenzhen University, Shenzhen, China

**Keywords:** exercise, Baduanjin, Qigong, mind-body exercise, osteoarthritis, postural stability, proprioception

## Abstract

**Background:** Knee osteoarthritis is a common disease affecting a large number of old individuals worldwide. This study aimed to explore the effects of Baduanjin Qigong in patients with knee osteoarthritis.

**Methods:** Fifty participants with knee osteoarthritis were randomly assigned to either an experimental group (*n* = 25) or a control group (*n* = 25). Participants in the experimental group received Baduanjin Qigong training for 12 weeks, with three sessions per week lasting 40 min per session. Participants in the control group did not receive any additional physical training. All of participants completed outcome (proprioception, postural stability, and functional ability) assessments at three time points (baseline, Week 8, 12).

**Results:** Proprioception and Western Ontario and McMaster Universities Osteoarthritis Index function were statistically improved at eighth and 12th week of the intervention in the Baduanjin Qigong group (*p* < 0.05), while the control group did not have any significant changes. For postural stability at the anterior-posterior direction with eyes closed, Baduanjin Qigong group showed significant improvement compared to controls after the 12 weeks of intervention (*p* < 0.05).

**Conclusions:** Regular Baduanjin Qigong practice helped the improvement of knee joint proprioception and postural stability, and reduction of pain, stiffness, and functional impairments of old adults with knee osteoarthritis. Well-designed randomized controlled trials with long-term assessment are needed. The trial was registered in Chinese Clinical Trial Registry (ChiCTR-IOR-16010042). URL: http://www.chictr.org.cn/hvshowproject.aspx?id=10550.

## Introduction

Knee osteoarthritis (KOA) is one of the leading causes of disability, and affects 10–15% of adults aged 60 years and older globally (1). KOA as a chronic joint disease primarily involves cartilage loss and abnormal bone growth (bone spurs or osteophytes) while joint space narrowing in the knee has occurred (2). Such this degenerative process can directly lead to pain, joint stiffness, and swelling (2). Furthermore, physical function deficits (e.g., muscle strength, postural stability, and proprioception) are also observed in patients with KOA (3, 4), which are associated with a higher incidence of falls in this population (5).

The potential mechanism of postural instability in KOA is complex in terms of the sensory-motor coupling (6). It is well-documented that muscle weakness and pain are associated with impaired postural control because pain inhibits muscle activation and torque (7, 8). In patients with symptomatic KOA, the sensory information that fed back to the central nervous system (CNS) might be affected by unexpected conditions like pain and rough roads (9). More likely, the CNS is adaptive by the pain sensitivity and proprioception to rebuild a postural control. It indicates that the pain sensitivity may limit the sensory input of information to the CNS, which affects the motor function output of postural control (10).

Despite non-steroidal anti-inflammatory drugs (NSAIDs) aimed at reducing pain and swelling, these first-line agents are not only unaffordable to those economically disadvantaged KOA patients, but their long-term use also causes adverse effects such as gastrointestinal bleeding and perforation (11, 12). Physical exercise training has been shown to have the beneficial effects for patients with KOA (13). Many studies reported that postural control, proprioception, and pain sensation were statistically improved after isokinetic muscle training exercises (14–16).

In addition to this conventional training method, KOA patients also attempted to seek complementary and alternative therapies such as Qigong exercises (17), which has driven researchers to investigate their therapeutic effects for KOA patients.

Qigong exercises can reduce pain, improve functional abilities (postural stability, muscular strength, flexibility) and help KOA patients maintain normal life activities (18–22). Baduanjin Qigong (BD), one of traditional Chinese Qigong exercise (Tai Chi, Wuqinxi, and Liuzijue) therapies (23, 24), has a great emphasis on mind-body integration; slow body movements along with musculoskeletal stretching should be coordinated with deep breathing, physical relaxation, and mental concentration, leading to a deep state of meditative therapy (25, 26). Of note, BD with mild exercise intensity has recently received substantial attentions from the research community. As the number of trials increase, researchers subsequently conducted several systematic reviews with meta-analytic method, suggesting that BD has therapeutic effects for individuals with medical conditions (depression, anxiety, insomnia, chronic obstructive pulmonary disease, musculoskeletal pain, and stroke) (27–31).

However, to date, there has been only one clinical trial attempting to investigate the feasibility and safety of BD in treating KOA (32). Results of this previous study (32) indicated that BD is a safe and feasible treatment option for KOA patients as well as effective to reduce pain, stiffness, and disability (leading to better quadriceps strength and aerobic ability). Poor postural stability performance and lower-limb proprioception were also reported in these KOA patients (33–35). Given that these poor functional abilities are associated with greater possibility of fall-related injuries and death (36), it has great value to determine whether BD therapy has the potential to improve these outcomes while reducing pain, stiffness, and disability. Previous study involved five instructor-led sessions per week, with each BD session lasting 60 min during an 12-week intervention period (37). Such training mode/regime is hard to be realized in our hospital setting, as a result of many kinds of factor influence (e.g., transportation of patients and time-starved clinicians/researchers). Moreover, such training mode/regime does not guarantee effectiveness after the completion of intensive supervised training sessions or experiments. Whether self-practice of BD would be beneficial to KOA patients is unknown. Thus, to investigate the therapeutic effects of BD for KOA patients, we conducted a randomized controlled trial in which a 12-week BD consisted of two phases (instructor-led training mode for first 4 weeks and home-based practice for the remaining of 8 weeks).

## Methods

### Study Design

This clinical trial was designed as a randomized, single-blind, two-arm parallel assignment. We reported the study procedures and results in accordance with the CONSORT checklist (38). All eligible individuals were randomly assigned to either an experimental BD or control group at a 1:1 ratio via PLAN sentences of the statistical software SAS 9.1. The experimental group received a 12-week BD training, whereas the control group maintained their unaltered lifestyle during the study period. Before initiating, all eligible participants were asked to sign their informed consent forms, which was conducted in accordance with the declaration of Helsinki and approved by the Medical Ethics Committee of the Affiliated Rehabilitation Hospital of Fujian University of Traditional Chinese Medicine (approval number: 2014KY-020-01). The trial was registered in Chinese Clinical Trial Registry (ChiCTR-IOR-16010042).

### Study Participants

Participants were recruited through advertisements and referral from their doctors of the Rehabilitation Hospital (Fujian, China) between January and December 2016. Participants were considered eligible if they: (1) were diagnosed with KOA according to criteria of the American College of Rheumatology (39), with radiographic grading of the severity between 2 and 3 (40) and knee pain of <5 on the 10-point Visual Analog Scale; (2) were aged between 50 and 80; (3) were able to independently ambulate without language problem in order to perform BD movements. We excluded those participants who: (1) suffered major diseases (cardiovascular, respiratory, or other musculoskeletal diseases) that required hospitalization; (2) had an implanted cardiac pacemaker; (3) were on medication affecting the musculoskeletal system, or proprioception and postural stability (e.g., anti–depressants, dopaminergic agents, and hypnotic) (41); (4) partook in regular exercise of more than three times per week; (5) fractured a bone within the past 12-months.

### Intervention Protocol and Control Condition

A 12-week intervention program was provided to KOA patients in the BD group, and it involved 3 sessions per week, with each session lasting 40 min (10 min for a warm-up and cool-down and 30-min for BD movements). The BD training regime was in line with Health Qigong-Baduanjin published by the Health-Qigong Management Center of the General Administration of Sport of China in 2003 (42). More specifically, this intervention program involved two phases. KOA patients were asked to attend group-based BD training for first 4 weeks at the Rehabilitation Hospital, administered by a certified instructor with at least of 5 years of teaching experience. In phase 2 (Week 4–12), KOA patients were asked to practice at home. To maximize adherence to the BD training program, they were required to record themselves during practice. In addition, a reminder phone call was made every 2 weeks to increase exercise adherence. In the meanwhile, KOA patients were required to return to the Rehabilitation Hospital once per month and to attend the group-based BD training in which KOA patients were gave the opportunities to ask questions about BD. KOA patients in the control group were informed to maintain their unaltered lifestyle while refraining from other supervised exercise training program.

To monitor their lifestyle, phone calls were made biweekly to remind these KOA patients not to involve in any extra physical exercise. After the completion of this study, KOA patients of the control group were provided with the same BD training program.

### Outcome Measures

We collected anthropometric data [e.g., body mass index (BMI)] (Table 1) at baseline because obesity is closely related to muscle weakness and poor balance (43). Assessments of postural stability, proprioception and self-reported function were taken at three time points: baseline (Week 0), Week 8 and post-intervention (Week 12). Proprioception and postural stability assessments occurred at the Fujian Key Laboratory of Rehabilitation Technology, China. We used the Western Ontario and McMaster Universities Osteoarthritis Index (WOMAC) to assess symptoms of KOA at Fujian Key Laboratory of Integrative Medicine on Geriatrics, China. Research assistants were blinded to group assignment completed all assessments. Although blinding of participants was unrealistic during exercise intervention, to minimize performance and detection biases, we urged participants not to disclose their allocation information during the study period. All KOA patients were given time to familiarize with the outcome measures 1 week prior to the actual assessment.

**Table 1:** Demographic data of participants.

**Characteristics**	**BD**	**CG**	***p***
	**(*n* = 25)**	**(*n* = 25)**	
Age, year Gender: females, %	64.48 ± 7.81 13(52)	63.08 ± 3.65 17(68)	0.412 0.333
Height, cm	163.28 ± 7.83	161.92 ± 7.56	0.535
Weight, kg	64.60 ± 9.19	64.20 ± 5.70	0.854
Body mass index, kg/m^2^	24.15 ± 2.47	24.56 ± 2.31	0.553

*BD, Baduanjin group; CG, control group*.

#### Primary Outcome Measures

To measure postural stability, the Prokin system (PK-252, Tecnobody, Italy) was used to quantify body sway. Barefooted, participants were instructed to stand in an upright reference position on the footboard and maintain that posture for 30 s with their eyes open, and then with eyes closed (task was created by the machine—open first, closed after). During tests with eyes open, fixed their view on a wall position 1.5 m in front. Variations in center of pressure (COP) position were quantified by anterior-posterior (AP) and medial-lateral (ML) displacements measured in millimeters (mm) (9).

To measure postural stability and proprioception at the knee, the Prokin system was used. This system employs an electronic transducer board mounted on a pivot to record foot movement. To measure proprioception, participants were seated on a chair adjusted to the horizontal position of the participants' thigh, allowing a knee joint angle of 110° and ankle plantar flexion of 10°. The affected foot was positioned at the center of the footboard while the other foot remained in a rest position. Participants were instructed to follow a predefined circular route with their foot. For right limb affected participants, clockwise movements were performed whereas anticlockwise movements were performed for left limb affected. Participants were instructed to perform five circular laps for each limb as fast as possible and to the best of their ability within a maximum allotted time of 60 s. Variables of interest were average trace error (ATE) in percentage (%) and test time execution (TTE) in seconds (s) (44).

#### Secondary Outcome Measures

The WOMAC is a patient-based assessment tool that involves 24 items within three subscales and has high reliability and validity (45–47). This is widely used for professionals to evaluate the condition of patients with knee or hip osteoarthritis (48). KOA patients were asked to indicate their level of pain (five items), joint stiffness (two items), and physical function (17 items) on a five-point Likert-scale, with zero representing no impairment to four representing severe impairment.

### Data Analysis

The demographic data of baseline characteristics were presented with descriptive statistics. Data normality was assessed using the Shapiro–Wilk test. Individual outcome measures were assessed using two-way repeated measures ANOVA with assessment interval serving as the within-subject factor and intervention group as the between-subject factor. If a significant interaction emerged, we used the simple effect tests. *Post-hoc t*-tests with Bonferroni correction were used following significant main effects. All statistical analyses were performed using SPSS 19 (IBM, New York, U.S.) using an alpha level of 0.05 to indicate a significant level.

## Results

**Figure 1:**
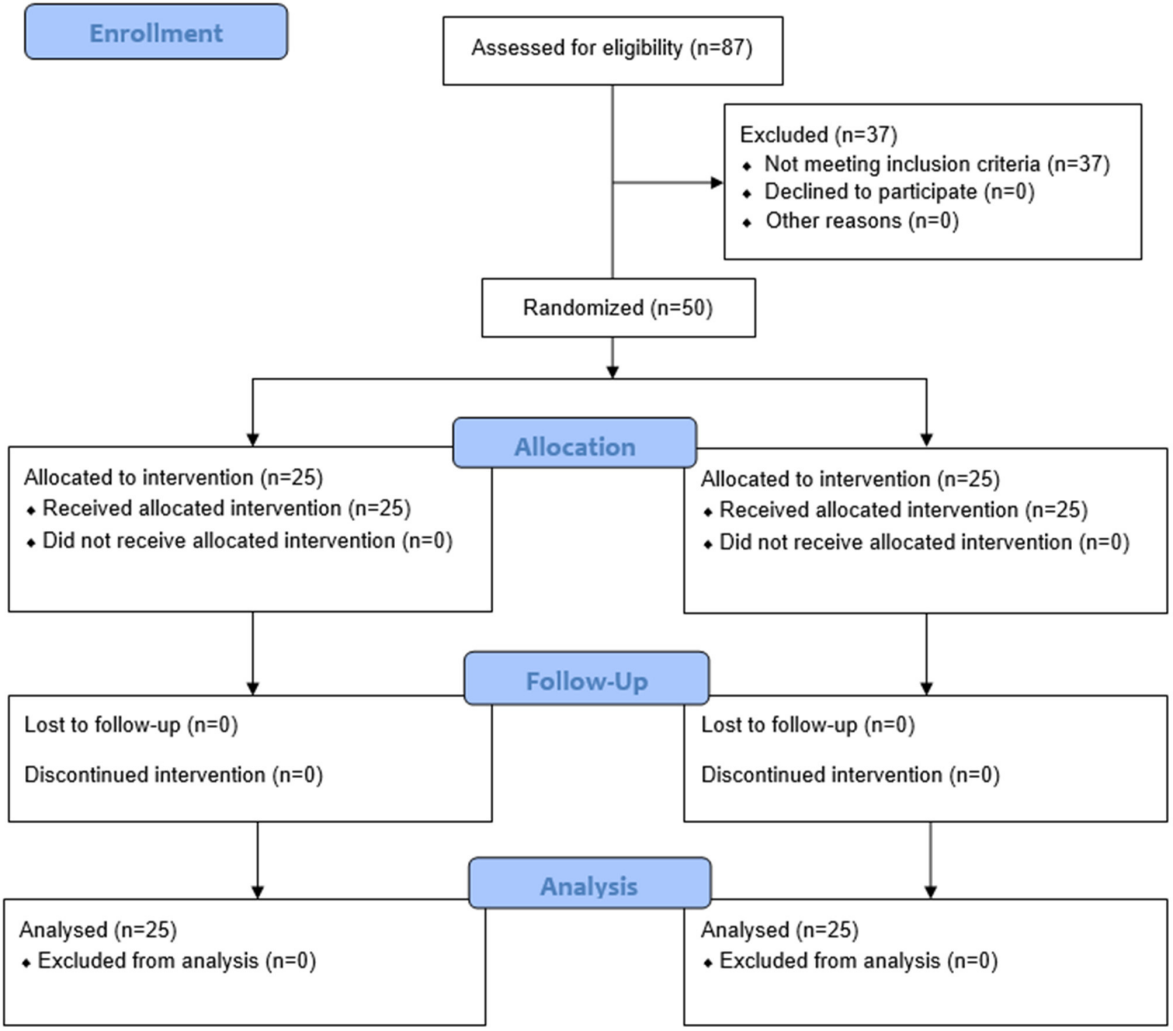
Flow chart of the progress of all procedure.

A total of 87 individuals who were interested in this study replied to our research staffs, 37 of which were excluded as they did not meet inclusion criteria. Fifty participants were finally included in this study and were randomly assigned to either the BD group (*n* = 25, female = 13, male = 12) or the control group (*n* = 25, female = 17, male = 8). The average age is 64.48 ± 7.81 for BD and 63.08 for the control group, respectively. Demographic information is presented in Table 1. Detailed information about study procedures are presented in Figure 1.

The primary outcomes (postural stability and proprioception) and secondary outcome (WOMAC) are presented in Table 2. The values of all variables were similar in the two groups at baseline (*P* > 0.05). No side effect was reported during the 12-week intervention period.

**Table 2:** Mean ± SD performance value of postural stability, proprioception, and WOMAC.

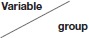	**Time point** **mean** **±** **SD**				**Condition** **effect**	**Time** **effect**	**Condition x time effect**		
**Postural stability** **(mm)**	**Baseline**	***P***	**Week 8**	**Week 12**	***P***	***P***	***P***	**F**	**η^2^**
**OPAP**									
BD	4.92 ± 0.91	0.184	4.67 ± 1.23	4.71 ± 1.54	0.754	0.588	0.440	0.760	0.016
CG	4.60 ± 0.76		4.56 ± 1.29	5.08 ± 2.00					
**OPML**									
BD	4.44 ± 1.12	0.223	4.04 ± 0.94	4.36 ± 2.12	0.850	0.301	0.373	0.931	0.019
CG	4.00 ± 1.38		4.16 ± 1.03	4.84 ± 2.09					
**CPAP**									
BD	6.28 ± 1.72	0.769	5.48 ± 0.96	5.44 ± 1.26	0.006^*^	0.217	0.035^*^	3.459	0.067
CG	6.16 ± 1.07		6.08 ± 1.12	6.64 ± 1.11					
**CPML**									
BD	5.96 ± 1.74	0.928	5.64 ± 1.58	5.56 ± 1.08	0.093	0.993	0.376	0.987	0.020
CG	5.92 ± 1.32		6.282.37	6.40 ± 1.61					
**Proprioception**									
**TTE (s)**									
BD	43.84 ± 10.84	0.500	38.52 ± 12.13	31.16 ± 6.81	0.023^*^	0.001^*^	0.029^*^	3.699	0.071
CG	41.96 ± 8.61		43.40 ± 11.84	39.64 ± 7.11					
**ATE (%)**									
BD	58.56 ± 17.99	0.093	37.60 ± 17.08	43.20 ± 20.64	0.001^*^	0.001^*^	0.144	1.977	0.040
CG	66.84 ± 16.12		57.56 ± 13.76	54.00 ± 12.37					
**WOMAC**									
**Overall**									
BD	31.94 ± 12.32	0.523	20.08 ± 9.95	14.52 ± 6.17	0.003^*^	0.001^*^	0.001^*^	19.572	0.290
CG	29.24 ± 11.41		30.16 ± 10.95	28.28 ± 8.40					
**Pain**									
BD	6.64 ± 2.74	0.528	2.88 ± 1.54	3.68 ± 6.07	0.015^*^	0.011^*^	0.357	0.988	0.020
CG	7.68 ± 9.34		6.48 ± 2.37	4.92 ± 1.41					
**Stiffness**									
BD	4.16 ± 2.14	0.757	2.04 ± 1.43	2.32 ± 1.38	0.001^*^	0.686	0.001^*^	31.248	0.390
CG	4.32 ± 1.44		7.00 ± 2.63	6.40 ± 2.04					
**Physical function**									
BD	20.84 ± 11.04	0.529	15.64 ± 8.87	9.52 ± 6.04	0.271	0.001^*^	0.005^*^	5.679	0.106
CG	19.08 ± 8.41		17.00 ± 8.71	16.24 ± 6.82					

*OP, open eyes; AP, anteroposterior; ML, mediolateral; CP, closed eyes; TTE, test time execution; ATE, average trace error; SD, standard deviation; BD, Baduanjin group; CG, control group; WOMAC, the Western Ontario and McMaster Universities Osteoarthritis Index*.

* *Denotes a significant effect (P < 0.05)*.

### Postural Stability

For postural stability, only a significant condition-by-time interaction effect for AP direction with eyes closed [*F*_(1,48)_ = 3.459, *p* = 0.035, η^2^ = 0.067; Table 2] was found. We ran a simple effect test to determine differences between two conditions at Week 8 and 12 (equivalent baseline). Results of the follow-up test indicate that BD group showed significantly greater improvement of AP direction at both Week 8 (95% CI −1.192 to −0.008, *p* = 0.047) and Week 12 (95% CI −1.876 to −0.524, *p* = 0.001; Tables 3A,B) than the control group. Neither group nor time effect was significant under other conditions (AP and ML with eye-open and ML with eye-closed) (*p* > 0.05; Table 2).

**Table 3A:** Results of outcome variables with *post-hoc* analysis over time in both groups.

**Variable**		**Pre vs. 8 post**		**8 post vs. 12 post**		**Pre vs. 12 post**	
		**BD**	**CG**	**BD**	**CG**	**BD**	**CG**
OPAP	*P*	0.557	0.900	1.00	0.303	0.656	0.247
(mm)	95% CI	−0.40 to 0.72	−0.61 to 0.69	−0.95 to 0.95	−1.54 to 0.50	−0.57 to 0.89	−1.32 to 0.36
OPML	*P*	0.187	0.632	0.513	0.223	0.868	0.215
(mm)	95% CI	−0.21 to 1.00	−0.84 to 0.52	−1.32 to 0.68	−1.80 to 0.44	−0.91 to 1.06	−2.20 to 0.52
CPAP	*P*	0.030	0.817	0.908	0.100	0.074	0.130
(mm)	95% CI	0.09 to 1.52	−0.62 to 0.78	−0.67 to 0.75	−1.24 to 0.12	−0.09 to 1.77	−1.11 to 0.15
CPML	*P*	0.476	0.519	0.853	0.827	0.266	0.286
(mm)	95% CI	−0.59 to 1.23	−1.50 to 0.78	−0.80 to 0.96	−1.24 to 1.00	−0.33 to 1.13	−1.39 to 0.43
TTE (s)	*P*	0.125	0.617	0.010^*^	0.151	0.001^*^	0.361
	95% CI	−1.59 to 12.23	7.31 to 4.43	7.47 to 17.89	−2.82 to 7.46	1.93 to 12.79	−1.47 to 8.99
ATE (%)	*P*	0.001^*^	0.029	0.277	0.331	0.001^*^	0.018
	95% CI	11.19 to 30.73	1.03 to 17.53	4.80 to −1.11	−3.85 to 10.97	8.03 to 22.69	2.44 to 23.24
Overall	*P*	0.001^*^	0.703	0.009^*^	0.152	0.001^*^	0.696
WOMAC	95% CI	6.68 to 15.96	−5.85 to 4.01	1.49 to 9.63	−0.74 to 4.50	13.38 to 20.38	−4.04 to 5.96
Pain	*P*	0.001^*^	0.541	0.530	0.003^*^	0.049	0.161
	95% CI	2.40 to 4.72	−2.80 to 5.20	−3.39 to 1.79	0.60 to 2.52	0.02 to 5.50	−1.18 to 6.70
Stiffness	*P*	0.001^*^	0.001^*^	0.436	0.321	0.001^*^	0.001^*^
	95% CI	1.20 to 3.05	−3.71 to −1.65	−1.01 to 0.45	−0.62 to 1.82	0.99 to 2.69	−2.91 to −1.26
Physical function	*P*	0.014^*^	0.300	0.002^*^	0.607	0.001^*^	0.176
	95% CI	1.18 to 9.23	−1.98 to 6.14	2.36 to 9.86	−2.25 to 3.77	8.23 to 14.41	−1.36 to 7.40

* *Denotes a significant effect (p < 0.017); BD, Baduanjin group; CG, control group; Pre, pretest; 8 post, 8th post assessment; 12 post, 12th post assessment*.

**Table 3B:** Results of outcome variables with *post-hoc* analysis between two groups.

**Variable**		**8 post**	**12 post**
OPAP (mm)	*P*	0.579	0.528
	95% CI	−0.59 to 0.92	−1.33 to 0.69
OPML (mm)	*P*	0.668	0.498
	95% CI	−0.68 to 0.44	−1.90 to 0.94
CPAP (mm)	*P*	0.047	0.001^*^
	95% CI	−1.19 to −0.01	−1.88 to 0.52
CPML (mm)	*P*	0.267	0.035
	95% CI	−1.79 to −0.51	−1.62 to −0.06
TTE (s)	*P*	0.157	0.001^*^
	95% CI	−11.70 to 1.94	−12.44 to 4.52
ATE (%)	*P*	0.001^*^	0.029
	95% CI	−28.74 to −11.14	−20.48 to −1.12
Overall	*P*	0.001^*^	0.001^*^
WOMAC	95% CI	−16.03 to −4.13	−17.95 to −9.57
Pain	*P*	0.001^*^	0.325
	95% CI	−4.73 to −2.47	−3.75 to 1.27
Stiffness	*P*	0.001^*^	0.001^*^
	95% CI	−6.16 to −3.76	−5.07 to −3.09
Physical function	*P*	0.587	0.001^*^
	95% CI	−6.36 to 3.64	−10.38 to −3.06

* *Denotes a significant effect (p < 0.025). 8 post, 8th post assessment; 12 post, 12th post assessment*.

### Proprioception at the Knee

For proprioception at the knee, we observed a significant condition-by-time interaction effect for TTE [*F*_(1,48)_ = 3.669, *p* = 0.029, η^2^ = 0.071; Table 2], but not for ATE [*F*_(1,48)_ = 1.977, *p* = 0.144, η^2^ = 0.040]. To determine differences between two conditions at Week 8 and 12 (equivalent baseline), we ran a simple effect test, indicating that BD group showed significantly greater reduction of TTE following the 12-week intervention than the control group (Tables 3A,B). In addition, results of condition effect indicated that BD group showed significantly better performance on the ATE at both Week 8 (95% CI −28.733 to −11.137, *p* = 0.001) and Week 12 (95% CI −20.476 to −1.124, *p* = 0.029) as compared to those in the control group (Tables 3A,B).

### WOMAC

Significant condition-by-time interaction effects were observed for overall WOMAC index [*F*_(1,48)_ = 19.572, *p* < 0.001, η^2^ = 0.290], stiffness domain [*F*_(1,48)_ = 31.248, *p* < 0.001, η^2^ = 0.390], and physical functioning domain [*F*_(1,48)_ = 5.679, *p* = 0.005, η^2^ = 0.106]. Results of follow-up simple effect test indicated that BD group showed significantly better performance on the three outcomes at Week 8 and 12 than those in the control group (*p* < 0.05). With regard to the pain domain, we observed significant group and time effect: (1) BD showed significantly better performance at Week 8 than the control group but not Week 12; (2) BD only showed significant improvement between baseline and Week 8 (*p* = 0.001) and between baseline and Week 12, as significant pain reduction was observed in the control group between Week 8 and 12 (*p* = 0.003).

## Discussion

The present study investigated the effects of BD on postural stability and knee proprioception on patients with symptoms of KOA aged over 60. Results indicated that a 12-week BD therapy improved proprioception at the knee joint, postural stability, and WOMAC function, as compared to the control group. To date, only one study investigated the effects of BD on WOMAC indexes, results of which are consistent with the present study. Furthermore, positive findings of our study are also supported by previous studies indicating the therapeutic effects among healthy subjects for lower extremity strength (49), as well as other Qigong exercise (Tai Chi) for postural stability (50), lower-limb proprioception (51, 52), and WOMAC function (51, 53–55) of KOA patients. Thus, BD as a mind-body exercise therapy may be effective for treating KOA patients.

In the present study, improved proprioception of knee could be attributed to increase in quadriceps muscle strength through the 12-week BD training. It has been reported that impaired proprioception of knee joint was related to quadriceps muscle weakness (56). Previous studies indicated that quadriceps strength had improved considerably after regular BD training (32, 57). Such positive effect on the muscle strength may provide protection against degeneration of knee proprioception. This positive effect observed in the knee proprioception may be partially explained by the feature of BD that has emphasis on precise control of both knee flexion and extension to maintain center of gravity while performing dynamic and symmetrical movements. The 12-week BD intervention may provide stimulation for developing better joint position sense, leading to decrease in the ATE and TTE. It is worth emphasizing that significant improvement of knee proprioception in the BD group was only observed at Week 12, as compared to the control group.

Our results demonstrated that only BD group showed a significant improvement of postural stability with eye-closed at the AP direction. Furthermore, such improvement started from Week 8 and continued till Week 12. It indicates that the minimum of 8-week BD training could trigger an initial response to postural stability. Positive effects on postural stability at the AP direction may be attributed to the local muscle control (local-control) including three BD movements that involve postural control while upper body is required to: (1) move in multiple directions (Movement 5, Sway the Head and Shake the Tail); (2) lean forward (Movement 8, Bouncing on the Toes), (3) or take weight off the heels (Movement 8, Bouncing on the Toes). Meanwhile, the higher central nervous sensory feedback cueing (central-control) may also contribute to this positive effect on postural stability. However, as we did not measure the central-control in present study, further work may include the measurement of control-of-gravity to investigate the performance of both local-and control-controls in patients with KOA (58, 59).

Postural stability is the ability to defend against a fall that is prevalent in aging individuals (60). Globally, falls are the second leading cause of accidental or unintentional injury deaths, and adults aged over 65 suffer the greatest number of fatal falls (61). Roughly 40 million serious falls occur yearly which requires medical attention (62). It is widely accepted that aging population with KOA showed lower postural stability, as compared to healthy age-matched controls (63–65). Thus, prioritizing fall-related research is critical so that we could establish effective policies to reduce risk.

Studies indicated that the quality of life among patients was largely affected by the knee pain (66, 67). Knee pain and stiffness would also cause psychological distress on KOA patients (1, 68). In the current study, we found with the practice of BD, the overall WOMAC index was improved, pain and stiffness were significantly decreased compared to the control group at Week 8. Physical function domain also showed statistically significant improvement across the three time points. Previous study done by An et al. (32) was supporting the current findings. They found significant reduction in the pain subscale after 8 weeks of BD training. Another study by An et al. (57) also showed long-term BD practice would improve WOMAC outcomes. With support from previous studies, BD exercise could be an effective intervention for relieving the syndromes on pain and stiffness and improving physical function in patients with KOA.

Although we've found BD as an exercise intervention, is beneficial to KOA patients, the current study still has some limitations. The participants of the study were recruited from one hospital location, leading to a sampling bias of the data, the generalizability of the application was limited. Also, in the current study we compared the benefits of BD to a not BD intervention group, it is not known the effectiveness of BD when comparing to other type of exercises among KOA patients. Further study may include other interventions such as strength training, aerobics, or complementary and alternative exercises (i.e., Tai Chi) to compare the effects of BD exercise in participants from multi-medical centers. On another aspect, from Week 4 to 12 of the present study was home-based, some functional benefits gradually disappear within the BD group, this might be due to the adherence to the BD exercise, since it was based on participants' self-awareness, although the program motivated participants with video recording during exercise and monthly check-up at the hospital, it was challenging to measure the effort participants paid to home-based exercise. However, even with home-based practice of BD, we still demonstrated the benefits of practicing BD on KOA patients. Finally, the sample size in each group was relatively small. This might not be enough to have the full training effects. In future studies, more advanced monitoring and supporting system could be applied to help patients keep practicing BD, and the self-motivation of BD exercise among large populations should be investigated.

## Conclusion

In summary, this study found that regular BD exercise improved proprioception of knee joint, pain, stiffness and disability in older adults with KOA. It indicates that a 12-week BD regime (3 sessions per week, with each session lasting 40 min) should be designed for KOA patients to gain positive effect of knee proprioception. Current findings indicate that the application of a BD program is an effective option in elderly with KOA, and home-based practice of BD could still benefit the population.

Although it is not highly recommended to practice at home without involving in domestic helpers or family members because the compliance of participants is difficult to control, it is a powerful scientific support to a good start of advocating home-based BD practice. Further work should conduct higher quality RCTs with larger sample size and long-term intervention period in patients with KOA to investigate the effectiveness of BD on postural stability (at the anterior-posterior direction with eyes closed) function. Findings of this future study may have beneficial effects in reducing fall risk for patients with KOA.

## Data Availability Statement

The datasets generated for this study are available on request to the corresponding author.

## Ethics Statement

The studies involving human participants were reviewed and approved by Local Ethics Committee (approval number: 2014KY-020-01). The trial was registered in Chinese Clinical Trial Registry (ChiCTR-IOR-16010042). The patients/participants provided their written informed consent to participate in this study.

## Author Contributions

JY: conceptualization. MS, WZ, and SC: data curation. JY and SC: funding acquisition. JY, WL, WZ, and SC: resources. YL, LZ, and MS: methodology. JY, MS, WL, WZ, LZ, and SC: writing—review and editing. All authors read and approved the final manuscript.

### Conflict of Interest

The authors declare that the research was conducted in the absence of any commercial or financial relationships that could be construed as a potential conflict of interest.
